# Knowledge, attitude and practice about the Personal Health Record among general population in Italy

**DOI:** 10.1093/eurpub/ckac131.185

**Published:** 2022-10-25

**Authors:** A Prinzivalli, G Scaioli, L Guastavigna, A Scacchi, G Lo Moro, F Bert, R Siliquini

**Affiliations:** 1 Department of Public Health Sciences, University of Turin, Turin, Italy; 2 A.O.U. City of Health and Science of Turin, Turin, Italy

## Abstract

**Background:**

The Personal Health Record (PHR) is an online tool containing a person’s health-related data, which can be shared with health professionals. This tool is widely used throughout Europe but is not very popular in Italy, despite its implementation being mandatory. This study aims to investigate knowledge, attitudes and practices (KAP) associated with the PHR among the general population in Italy, to identify the main obstacles in the usage of PHR and to establish strategies that lead to their resolution.

**Methods:**

A cross-sectional survey was designed and shared both online and on paper (in the waiting rooms of the outpatient clinics) to an opportunistic sample of people aged ≥18 years. Factors associated with a higher knowledge and willingness to activate the PHR were investigated through multivariable logistic regression analyses.

**Results:**

Preliminary results of the on paper survey (243 answers) showed that the median age of the participants was 45, 46% were women, 9% worked in healthcare and 68% had chronic diseases. Only 31% heard about the PHR, of which 22% showed a good knowledge of it. The 66% declared the willingness to activate the PHR and 92% agreed that the PHR is a useful tool for physicians to share information. Multivariable logistic regression models showed that those who were employed and those who were visited by a doctor in the last three months had a higher likelihood of having heard about PHR (OR 2.56, 95% CI 1.12 - 5.82 and OR 2.67, 95% CI 1.20 - 5.90 respectively). The likelihood of being willing to activate the PHR is lower for those who live alone, and is higher for those who are employed (OR 0.35, 95% CI 0.14 - 0.88 and OR 2.35, 95% CI 1.06 - 5.24).

**Conclusions:**

The results confirm the low level of knowledge and diffusion of the PHR in Italy, although a relatively high interest in this tool was highlighted, along with a favorable perception of its usefulness. Promotional interventions are needed to increase knowledge and awareness of this tool.

**Key messages:**

• Awareness about Personal Health Record among the Italian population is low. Despite this, a high level of willingness to activate this tool and a high perception of its usefulness were registered.

• Identifying factors that lead to lower awareness on Personal Health Record may help Public Health professionals to implement targeted promotional interventions aimed to increase the usage of this tool.

